# Bilateral localization of necrotizing sialometaplasia: a case report

**DOI:** 10.4076/1757-1626-2-9068

**Published:** 2009-09-08

**Authors:** Iwona Niedzielska, Tomasz Janic, Jarosław Markowski

**Affiliations:** 1 Clinic of the Maxillofacial Surgery Department Medical University of Silesia in Katowice, Francuska St. 20-24, Poland

## Abstract

**Introduction:**

Necrotizing sialometaplasia is a rare disease first described in 1973. It involves salivary gland in the oral cavity.

**Case presentation:**

We present a 32-year-old, white, male with rare bilateral localization of necrotizing sialometaplasia.

**Conclusion:**

Clinically and histologically necrotizing sialometaplasia may mimic malignant lesions. In the above presented case, the patient's history was suggestive of a malignant process. Thus, knowledge and experience of both clinicians and histopathologists are essential to establish a correct diagnosis, which helps avoid radical surgery.

## Introduction

Necrotizing sialometaplasia is an inflammatory process first described by Abrams et al. in 1973. The condition most frequently involves minor salivary glands in the hard palate region [1]; other anatomical locations, i.e., the oral cavity, minor salivary glands, and upper respiratory tract have also been observed [2]-[5]. An incidence of 0.03 to 0.063% has been reported [6,7]. Although the ethiopathogenesis has not been fully elucidated, the lesion is usually regarded as resulting from an ischemic event [1,8]. Predisposing factors include trauma, smoking, alcohol abuse [2,8], and recurrent vomiting [9]. A patient usually presents with ulceration, and, although more rarely, swelling of the mucous membrane [2]. Histologically, there is necrosis of the minor salivary gland structures; however, lobular architecture is maintained. Squamous metaplasia is seen within the mucinous acini and ducts, which makes the condition similar to malignancy; mucin extravasation and inflammatory reaction are also present [1]. Necrotizing sialometaplasia is a self-healing disease usually resolving within a period of 3 to 12 weeks [2].

## Case presentation

We report a case of 32-year-old white male who was seen in the Outpatient Clinic of the Maxillofacial Surgery Department in Katowice in April 2007 with a recurrent bilateral palate swelling which had appeared three years before. On the day of the examination the patient also complained of jelly-like nasal discharge and general malaise. The history was suggestive of neoplastic disease including a nondiagnosed palpable anal tumour and recent diagnostic procedures of lung lesions (no medical documentation available).

**Figure 1:**
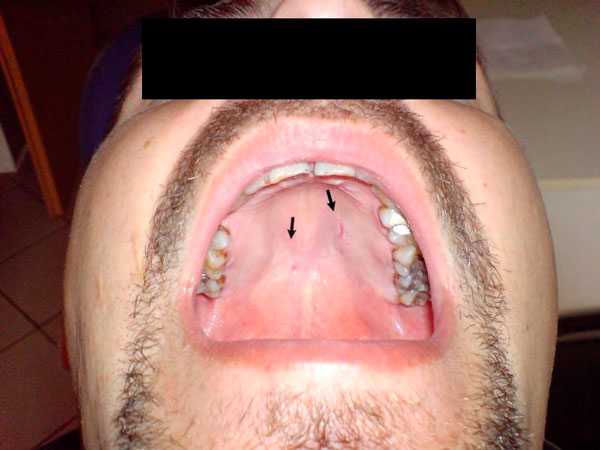
**Symmetrical affection in the area of the maxillary tuberosities**.

The clinical examination revealed a soft-elastic symmetrical affection in the area of the maxillary tuberosities; the covering mucous membrane was of normal appearance (Figure 1). The results of laboratory blood and urine tests were within normal range. Pantomogram and sinus X-ray did not detect any abnormalities. Facial CT showed a fluid-filled polypoid mucous membrane thickening of the medial maxillary sinus wall. The chest X-ray was normal. Rectoscopy revealed an anal varix to be first treated with anti-inflammatory agents, and then a surgical procedure. Fine needle aspiration biopsy showed 'epithelial nests and myxoid connective tissue suggesting a mixed tumor' *(44035/BC Właszczuk).* Therefore an incisional biopsy was performed with a diagnosis of necrotizing sialometaplasia (34685/K Pająk). The patient was hospitalized, and the lesion excised on the left side. Histologic features of the lesion were consistent with necrotizing sialometaplasia.

## Conclusion

The location of necrotizing sialometaplasia was typical as most of the lesions were seen on the palate [2,10]. However, bilateral symmetrical affection occurs rarely; Brannon et al. reported a 12% incidence [2]. The patient gave a 3-year history of symptoms recurrence, which is in accordance with the observations of Arguelles et al. [11]; however, our follow-up examinations did not confirm the patient's complaints.

Clinically and histologically necrotizing sialometaplasia may mimic malignant lesions. In the above presented case, the patient's history was suggestive of a malignant process. Thus, knowledge and experience of both clinicians and histopathologists are essential to establish a correct diagnosis which helps avoid radical surgery.

## Abbreviation

CT: computed tomography.

## Consent

Written informed consent was obtained from the patient for publication of this case report and accompanying image. A copy of the written consent is available for review by the Editor-in-Chief of this journal.

## Competing interests

The authors declare that they have no competing interests.

## Authors' contributions

IN involved in clinical examination, fine needle aspiration biopsy, writing the manuscript. TJ involved in literature review, writing the manuscript, clinical examination. JM involved. in writing the manuscript, clinical examination. All authors read and approved the final manuscript.
